# Supplementary material for: Global hypo-methylation in a proportion of glioblastoma enriched for an astrocytic signature is associated with increased invasion and altered immune landscape
Source: eLife. 2022 Nov 22;11:e77335. doi: 10.7554/eLife.77335 (PMC9681209; doi:10.7554/eLife.77335)
Supplement: Figure 2—source data 1. [file elife-77335-fig2-data1.zip › Figure_2_source_data_1/Figure_2B/homerResults/motif16.similar.html]

motif16

## Information for motif16

T
A
C
G
C
T
A
G
A
G
C
T
T
C
G
A
G
T
A
C
T
G
A
C
C
G
A
T
T
G
C
A
C
G
T
A
C
T
A
G
  
Reverse Opposite:  

G
A
T
C
G
C
A
T
A
C
G
T
G
C
T
A
A
C
T
G
A
C
T
G
A
G
C
T
T
C
G
A
G
A
T
C
A
T
G
C
  

|  |  |
| --- | --- |
| p-value: | 1e-59 |
| log p-value: | -1.361e+02 |
| Information Content per bp: | 1.361 |
| Number of Target Sequences with motif | 82.0 |
| Percentage of Target Sequences with motif | 3.55% |
| Number of Background Sequences with motif | 0.9 |
| Percentage of Background Sequences with motif | 0.25% |
| Average Position of motif in Targets | 94.4 +/- 55.8bp |
| Average Position of motif in Background | 184.0 +/- 0.0bp |
| Strand Bias (log2 ratio + to - strand density) | -0.4 |
| Multiplicity (# of sites on avg that occur together) | 1.02 |
| Motif File: | file (matrix) reverse opposite |

### Similar de novo motifs found

|  |  |  |  |  |  |  |  |
| --- | --- | --- | --- | --- | --- | --- | --- |
| Rank | Match Score | Redundant Motif | P-value | log P-value | % of Targets | % of Background | Motif file |
| 1 | 0.625 | G T C A A T C G A T C G A C G T C G T A A C G T | 1e-28 | -64.619437 | 29.51% | 19.98% | motif file (matrix) |
| 2 | 0.675 | A C T G A C G T C G T A A G T C A G T C A C T G C G T A A C G T | 1e-17 | -40.067822 | 2.38% | 0.78% | motif file (matrix) |
